# Headache Attributed to Infection: A Clinical and Pathophysiological Overview

**DOI:** 10.1007/s11916-026-01473-9

**Published:** 2026-04-08

**Authors:** Márk Kozák, Laszlo Mechtler, Christopher Ralyea, Viktor Bencs, Rebeka Hodossy-Takács, István Várkonyi

**Affiliations:** 1 Department of Infectology, Faculty of Medicine, University of Debrecen, Bartok Bela Street 2-26, Debrecen, 4031 Hungary; 2 DENT Neurologic Institute Buffalo, Buffalo, NY USA; 3 Department of Neurology, Faculty of Medicine, University of Debrecen, 22 Móricz Zsigmond krt, Debrecen, 4032 Hungary; 4 Division of Clinical Laboratory Sciences, Department of Laboratory Medicine, Faculty of Medicine, University of Debrecen, MTA-DE Lendület “Momentum” Hemostasis and Stroke Research Group, Debrecen, Hungary

**Keywords:** Headache, Infection, Secondary headache, Meningitis, Encephalitis, ICHD-3, Postinfectious headache, Red flags

## Abstract

**Purpose of Review:**

This narrative review summarizes current knowledge on infection-related headaches, with focus on classification, clinical presentation, and underlying pathophysiology. As infections increasingly present with neurological symptoms, recognizing headache as a potential red flag remains essential.

**Recent Findings:**

Headaches attributed to infection range from typical forms such as bacterial meningitis to more subtle parasitic, fungal, or postinfectious entities. The ICHD-3 provides structured diagnostic subtypes, including acute, chronic, and persistent forms. Emerging data on CSF biomarkers and neuroimaging aid in distinguishing secondary headaches from primary mimics. Postinfectious headache, particularly after viral illness or in immunocompromised states, is gaining attention as a long-term sequela.

**Summary:**

Infectious headaches are clinically diverse and require a structured, classification-based diagnostic approach. Integrating clinical phenotype, imaging, and CSF analysis can support early diagnosis. Further research is warranted to clarify pathogen-specific mechanisms and optimize management.

## Introduction

Headaches are estimated to affect more than 90% of the global population at some point during their lifetime and represent one of the most common complaints encountered in primary care, urgent care settings, emergency departments, and neurology clinics. Although the majority of headaches are due to primary disorders, a significant minority are secondary, with infectious etiologies among the most important causes [1]. Headache is a nearly universal symptom across a wide range of infectious diseases involving the central nervous system (CNS), including meningitis, encephalitis, and brain abscesses. In some cases it may be the first or only clinical manifestation of a potentially life-threatening condition [2]. Epidemiological data suggest that up to 60% of individuals experience headache in association with an infectious illness at some point in their lives [2, 3]. Headache is a common feature of systemic infections, often related to factors such as inflammation, dehydration, or metabolic imbalance. Despite its frequency, identifying clinical patterns suggestive of an infectious origin can be challenging. It may present as an isolated symptom, as a key sign of a severe infection, or be entirely absent. Underlying mechanisms include meningeal irritation, elevated intracranial pressure, and activation of the trigeminovascular system [4]. This article provides an overview of headaches attributed to systemic and/or intracranial infection, with particular emphasis on diagnostic red flags and classification. Given the broad scope of this topic, specific treatment protocols are beyond the scope of this review.

### Classification and Diagnostic Criteria

**Fig. 1:**
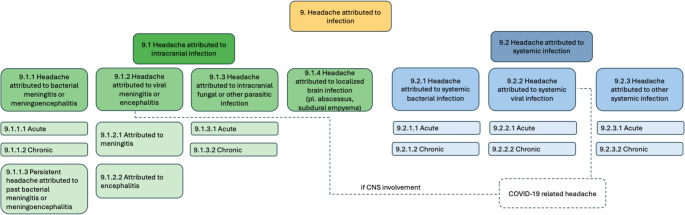
ICHD-3 classification of headache attributed to infection

The International Classification of Headache Disorders, 3rd edition (ICHD-3), classifies headache attributed to infection within Chap. 9, distinguishing between **“9.1** Headache attributed to intracranial infection” and **“**9.2 Headache attributed to systemic infection.**”** It further differentiates acute and chronic headache attributed to active infection, and, where applicable, persistent headache attributed to past infection (Fig. 1.). This classification framework highlights the importance of the temporal association between headache onset and the infectious process and considers the evolution or persistence of symptoms after resolution of the infection.

According to the ICHD-3, headache attributed to infection is diagnosed when a headache occurs in the presence of an active infection or its sequelae known to be capable of causing headache. Evidence of causation must be demonstrated by at least two of the following:

- The headache develops in close temporal relation to the onset of the infection;
- The headache significantly worsens in parallel with worsening of the infection and/or improves or resolves with improvement or resolution of the infection;
- The headache has characteristics typical for the underlying infectious disorder.

Finally, the diagnosis requires that the headache is not better accounted for by another ICHD-3 diagnosis [5].

The major subtypes of headache attributed to infection in ICHD-3 include headache attributed to intracranial infection (e.g., bacterial meningitis or meningoencephalitis; viral meningitis or encephalitis; intracranial fungal or parasitic infection; and localized brain infection such as abscess or empyema) and headache attributed to systemic infection (systemic bacterial, viral, or other systemic infection), each further categorized into acute or chronic forms and, where applicable, persistent forms depending on the temporal profile [5].

Of note, distinguishing between a primary headache exacerbated by infection and a true secondary infectious headache remains challenging. In such cases, dual coding (e.g., migraine and headache attributed to infection) may be appropriate [1].

These diagnostic categories are not only clinically relevant but guide therapeutic decision-making and imaging strategies. A lumbar puncture is often necessary to confirm intracranial infections, whereas systemic infections may be diagnosed through serologic, microbiologic, or imaging findings [6, 7].

Overview of the International Classification of Headache Disorders, 3rd edition (ICHD-3), Chap. 9, illustrating the hierarchical classification of headache attributed to infection. The classification distinguishes headache attributed to intracranial infection (9.1) from headache attributed to systemic infection (9.2). Within each category, headache is further classified according to its temporal relationship with the infectious process as acute or chronic headache attributed to active infection and, where applicable, persistent headache attributed to past infection. Emerging or clinically recognized entities, such as COVID-19–related headache, are not depicted as distinct diagnostic categories but are mapped onto existing ICHD-3 core diagnoses based on the underlying infectious mechanism.

### Pathophysiology of Headache Attributed to Infection

Headache in the context of infection arises from complex interactions between host immune responses, microbial toxins, vascular and meningeal involvement, and the activation of pain-generating pathways. Although clinical manifestations are often nonspecific, the underlying pathophysiological mechanisms vary substantially depending on the infectious agent and its anatomical localization.

The principal anatomical pain generators include the meninges (particularly the dura mater), large intracranial vessels, cranial nerves (notably trigeminal afferents), and pain-sensitive perivascular structures. These structures can be activated by mechanical traction (e.g., from raised intracranial pressure), chemical irritants, or inflammatory mediators [2].

#### Cytokine-Mediated Meningeal Irritation

Bacterial and viral pathogens induce a cascade of pro-inflammatory cytokines tumor necrosis factor-α, interleukin (IL)−6, and IL-1β within the cerebrospinal fluid (CSF) and meningeal vasculature. These cytokines increase blood–brain barrier permeability and stimulate nociceptive pathways, resulting in diffuse or pulsatile pain. Matrix metalloproteinases (e.g., MMP-9) further amplify leukocyte recruitment and vascular dysregulation, thereby contributing to headache severity [8]. This immune response causes blood-brain barrier disruption, vascular dysregulation, vasculitis and even occlusion of blood vessels which can all individually cause vasogenic brain oedema, loss of cerebrovascular autoregulation, and eventually increased intracranial pressure [2].

#### Raised Intracranial Pressure (ICP)

Increased ICP is a common pathogenic mechanism seen in bacterial meningitis, tuberculous meningitis, cryptococcal meningitis, and encephalitis. Edema formation, inflammatory exudate accumulation, and impaired CSF resorption at the arachnoid granulations contribute to pressure elevation. The resultant meningeal traction activates stretch-sensitive trigeminal afferents, leading to headache. In cryptococcal meningitis, fungal polysaccharides may physically obstruct CSF pathways, while tuberculous exudates can cause fibrin-mediated obstruction and chronic hydrocephalus [2, 6].

#### Direct Pathogen Effects and Toxin Release

Some organisms produce toxins (e.g., pneumolysin, streptolysin O, alpha-hemolysin) that directly activate nociceptors or potentiate inflammation [6]. In bacterial meningitis, lipopolysaccharide and peptidoglycan activate Toll-like receptors on meningeal macrophages and microglia, resulting in the release of prostaglandins and nitric oxide, which are key pain mediators [1, 2, 6].

#### Trigeminovascular System Activation

Key mediators (CGRP, substance P, neurokinin A) are released from trigeminal neurons in response to inflammation and play a central role in both primary and secondary headaches. The overlap in pathophysiology may explain why infection-related headaches can mimic migraine in some patients [2, 9].

#### Additional Contributors

Systemic factors, including fever, dehydration, electrolyte imbalance, and metabolic derangements may exacerbate headache during systemic infections even in the absence of direct central nervous system (CNS) involvement. These influences further blur the clinical distinction between primary and secondary headache presentations [1, 2, 9].

### Clinical Syndromes of Infection-Associated Headache

Headaches secondary to infections of the CNS can present with varied intensity, localization, and accompanying features depending on the causative pathogen and site of involvement. They may occur in isolation or alongside systemic and neurological symptoms, such as fever, meningismus, or altered consciousness [2].

These shared pathways provide a mechanistic scaffold for interpreting the syndrome-specific phenotypes described below.

#### Bacterial Meningitis

Headache is often the earliest and most prominent symptom in bacterial meningitis. It is typically severe, diffuse or occipitonuchal, and associated with photophobia, phonophobia, nausea, and neck stiffness [1]. Patients often describe a worsening of headache during sudden movements, particularly with rapid side-to-side head turns (2–3 times per second), known as jolt accentuation. To reduce discomfort, they may instinctively assume a flexed posture, due to reflex muscle spasms in the neck and spine that serve to shield the head and neck [10]. The typical symptom triad of bacterial meningitis includes fever, neck stiffness, and altered mental status; however, this combination is missing in up to half of all meningitis cases [11]. In infants and young children, headache is often not reported; rather, the clinical picture may include fever, fontanelle bulging, somnolence, altered consciousness, and seizures [12].

In bacterial meningitis, headache primarily reflects direct inflammatory involvement of the meninges, leading to sensitization of meningeal nociceptive pathways. This infectious meningeal inflammation underlies the characteristic association with neck stiffness, photophobia, and phonophobia, and may partly account for migraine-like headache features observed in some patients [1].

Disturbance of cerebrospinal fluid (CSF) dynamics with secondary elevation of intracranial pressure represents an additional mechanism contributing to headache in bacterial meningitis. This typically results in posture- and Valsalva-sensitive pain that worsens with coughing, bending forward, or lying flat and is often most pronounced in the morning. In bacterial infections associated with inflammatory obstruction of cerebrospinal fluid pathways, such as *Mycobacterium tuberculosis*, this mechanism may contribute to severe or persistent headache phenotypes. These are typically dominated by features of intracranial hypertension. In tuberculous meningitis, headache is commonly described as diffuse (holocephalic, ~ 60%) and throbbing (48.4%), with varying levels of severity ranging from mild (23.2%) to moderate (23.2%), severe (36.8%), or even intolerable (16.8%) [2].

In suspected bacterial meningitis, lumbar puncture (LP) is essential to obtain cerebrospinal fluid (CSF) for analysis, including cell count, glucose and protein levels, Gram stain, and culture. Typical CSF findings include low glucose concentration, elevated protein, and neutrophil-predominant pleocytosis. Because CSF glucose reflects serum glucose, the CSF-to-serum glucose ratio is a more reliable diagnostic marker than absolute CSF glucose values. Microbiological confirmation is achieved by identification of the causative pathogen on Gram stain or culture [8, 11]. A non-contrast computed tomography (CT) scan should precede LP in patients at increased risk of cerebral herniation, such as those with papilledema, new-onset seizures, focal neurological deficits, or immunosuppression. LP should be deferred in clinically unstable patients or in the presence of significant coagulopathy; however, empirical antibiotic therapy must not be delayed. Blood cultures are recommended, as concurrent bacteremia is present in approximately 53% of cases [13–15]. Multiplex polymerase chain reaction (PCR) assays performed directly on CSF provide a rapid alternative diagnostic approach, demonstrating high sensitivity and specificity (> 90%) for common bacterial pathogens (e.g., *S. pneumoniae*, *N. meningitidis*, *H. influenzae*, *S. agalactiae*), even when conventional cultures are negative [16].

#### Viral Meningitis

Viral meningitis is a common cause of secondary headache and is classified in the ICHD-3 under headache attributed to viral meningitis or encephalitis. The clinical onset is typically acute or subacute and is often preceded by a flu-like prodromal phase. The most frequent symptoms include headache, fever, and neck stiffness. Headache is usually moderate to severe in intensity, commonly frontal, retro-orbital, or holocranial, and is frequently accompanied by nausea, photophobia, and malaise. These features reflect meningeal irritation and the associated inflammatory response [1]. The ICHD-3 describes the headache phenotype in viral meningitis as holocranial or nuchal, often associated with neck stiffness, clinically overlapping with bacterial meningitis but generally following a milder course [5]. Compared with bacterial meningitis, viral meningitis generally follows a milder clinical course [2].

In viral meningitis, cerebrospinal fluid (CSF) typically demonstrates a mild to moderate pleocytosis (approximately 80–100 cells/µL) with lymphocytic predominance (> 80%). In the early phase of disease, however, neutrophil-predominant pleocytosis may be observed before evolving toward a lymphocytic pattern. Glucose and protein concentrations are generally within normal limits [17]. Importantly, CSF lymphocytosis accompanied by headache is not specific to viral meningitis and may also be seen in partially treated bacterial meningitis, *Mycoplasma pneumoniae*, *Listeria* spp., brucellosis, spirochetal and rickettsial infections, tuberculosis, as well as fungal or parasitic diseases [18]. Non-infectious causes should likewise be considered, including drug-induced aseptic meningitis (e.g., NSAIDs, antibiotics, immunosuppressants, intravenous immunoglobulins, chemotherapeutic agents), chemically induced meningitis, and meningitis associated with systemic inflammatory or infiltrative conditions such as vasculitis, sarcoidosis, connective tissue diseases, or meningeal carcinomatosis [19]. Polymerase chain reaction (PCR) analysis of CSF remains the diagnostic gold standard for confirming viral meningitis, allowing sensitive and specific detection of viral DNA or RNA [19–21].

#### Encephalitis

In contrast to meningitis, which involves inflammation of the meninges, encephalitis refers to inflammation of the brain parenchyma itself. While the process is often diffuse, it may also localize to specific regions, such as the medial temporal lobes in cases of herpes simplex encephalitis. Clinically, encephalitis usually presents as an acute febrile illness characterized by headache along with varying degrees of altered mental status, seizures, and/or focal neurological deficits [22].

Encephalitis can result from a primary infection caused by viruses, bacteria, fungi, or parasites, or may develop secondarily following a prior viral illness or vaccination. While the majority of encephalitis cases stem from primary viral infections, the causative pathogen can be definitively identified in only a subset of patients [22, 23]. Common viral agents include herpes simplex virus, other herpesviruses (such as varicella-zoster and Epstein-Barr), adenoviruses, influenza A virus, enteroviruses, and various arboviruses (e.g., Japanese encephalitis virus, St. Louis encephalitis virus, and West Nile virus). Nonviral infectious encephalitis can be caused by a range of pathogens, including bacteria (such as *Mycobacterium tuberculosis*,* Mycoplasma pneumoniae*,* Listeria monocytogenes*,* Borrelia burgdorferi*, *Leptospira* spp., *Brucella* spp., *Legionella* spp., *Tropheryma whipplei*, and *Treponema pallidum*), rickettsiae (e.g., *Rickettsia rickettsii*,* Coxiella burnetii*, and *Ehrlichia* spp.), fungi (e.g., *Cryptococcus* spp., *Aspergillus*, and *Coccidioides*), and parasites (e.g., *Plasmodium falciparum*,* Toxoplasma gondii*, or *Trypanosoma* spp.) [22]. A comprehensive discussion of these etiologies is beyond the scope of this article.

Although encephalitis most often presents with focal neurological deficits and cognitive disturbances, headache may occur early, particularly in the presence of meningeal involvement or associated cerebral vasculitis, and in some cases may represent the initial or sole clinical manifestation. The headache phenotype reflects combined parenchymal and meningeal involvement and should be interpreted within the general pathophysiological framework outlined in Sect. 2.2 [1].

Encephalitis-related headache is typically diffuse, often frontal or retro-orbital, severe or extremely intense, and may present as throbbing or pressure-like pain, sometimes accompanied by neck stiffness when meningeal irritation is present [2].

In viral encephalitis, lumbar puncture may reveal elevated opening pressure. Cerebrospinal fluid analysis typically shows lymphocytic pleocytosis, a mild increase in protein, and normal glucose levels. While brain magnetic resonance imaging (MRI) can appear normal, pathogen-specific changes, particularly in herpes simplex virus (HSV) encephalitis are frequently observed. Electroencephalography (EEG) abnormalities are observed in over 80% of cases, most commonly diffuse slowing and/or focal epileptiform discharges. In selected patients, continuous EEG monitoring is required to detect non-convulsive status epilepticus [23].

#### Brain Abscess

Brain abscesses are localized intracerebral infections characterized by a collection of pus and inflammatory cells, most often caused by bacteria or fungi. They represent a serious medical condition with significant risk for neurological sequelae and mortality, even with appropriate treatment. Mortality has declined over recent decades but remains notable. Abscesses may arise secondarily after neurosurgical procedures or primarily via hematogenous spread from distant infections, such as sinusitis, otitis, dental or pulmonary infections [24].

Headache is the most frequently reported initial symptom in brain abscesses. Other common clinical signs include confusion, altered mental status, nausea, vomiting, focal neurological deficits, fever, and seizures. Although the classic triad of headache, fever, and focal neurological signs is well known, it is uncommon at the time of presentation. From a headache perspective, brain abscess typically manifests as a subacutely evolving space-occupying lesion, consistent with the general mechanisms discussed in Sect. 2.2 [1].

Headache associated with brain abscess is reported in approximately 49–81% of cases and is usually progressive and localized. It is commonly exacerbated by coughing or Valsalva maneuvers, reflecting increased intracranial pressure and local mass effect [25].

Radiologic imaging plays a crucial role in diagnosis. Contrast-enhanced MRI is the modality of choice, typically revealing ring-enhancing lesions with surrounding edema and mass effect. CT scan may be used as an initial screening tool, especially in acute settings. Diffusion-weighted imaging (DWI) helps differentiate abscess from necrotic tumors. In uncertain cases, stereotactic aspiration may provide both diagnostic material and therapeutic benefit [26].

#### Subdural Empyema

Subdural empyema is a rare, but life-threatening intracranial infection characterized by purulent collection between the dura mater and arachnoid mater. It typically presents with severe, often lateralized headache, fever, focal neurological deficits, and seizures, and constitutes a neurosurgical emergency. In infants, it most commonly complicates meningitis, whereas in older children and adults it usually results from contiguous spread of sinusitis, otitis media, or mastoiditis, particularly via the frontal or ethmoid sinuses. Intracranial extension may occur through venous thrombophlebitis or direct bony erosion, while hematogenous spread or post-traumatic and post-surgical cases are less frequent. Causative organisms generally reflect the source and include anaerobes, *Streptococcus spp.*,* Staphylococcus spp.*,* Haemophilus influenzae*,* Streptococcus pneumoniae*, and gram-negative bacilli [1, 6]. The headache phenotype reflects combined meningeal inflammation and local mass effect [2, 6].

MRI with gadolinium contrast is the preferred modality for diagnosing intracranial subdural empyema. Contrast-enhanced MRI clearly delineates even small or interhemispheric collections, typically appearing as crescent-shaped hypointensities on T1-weighted images. DWI offers superior sensitivity and should be routinely included [27].

####  Headache Attributed to Intracranial Fungal or Parasitic Infection

Intracranial fungal or parasitic infections represent a rare but clinically relevant cause of secondary headache, categorized as ICHD-3 9.1.3. This diagnosis should be considered in immunocompromised patients and those exposed to endemic pathogens. The pathogenesis is typically related to inflammatory responses within the meninges or brain parenchyma, with or without associated mass effect or increased intracranial pressure [5, 6].

Cryptococcus neoformans is the most frequently implicated fungal pathogen in immunocompromised hosts, especially in HIV/AIDS patients. Its tendency to accumulate in the arachnoid granulations and obstruct CSF outflow can lead to raised intracranial pressure and chronic daily headache. Unlike bacterial meningitis, the CSF profile may reveal mild lymphocytic pleocytosis with elevated opening pressure and positive cryptococcal antigen testing. Other fungal agents, such as *Aspergillus* or *Candida* species, may cause cerebral abscesses or vasculitic complications, particularly in neutropenic or post-transplant patients. Invasive fungal infections may produce focal neurological deficits, seizures, or altered mental status, depending on the site of involvement [2, 5, 6].

Among parasitic infections, neurocysticercosis is the most common parasitic infection of the central nervous system worldwide and a frequent cause of secondary headache, particularly in endemic regions [28]. The pain may be associated with cyst degeneration, perilesional edema, or hydrocephalus due to ventricular obstruction. Other parasitic infections, including echinococcosis and toxoplasmosis, are less common but may mimic space-occupying lesions with raised intracranial pressure. Neuroimaging plays a central role in diagnosis, with characteristic findings such as cystic lesions, calcifications, perilesional edema, or hydrocephalus depending on the stage of the parasite and host immune response. Serological tests (e.g., enzyme-linked immunoelectrotransfer blot for Taenia solium) and CSF analysis may support the diagnosis but lack sufficient sensitivity or specificity on their own [1, 2, 5, 6].

#### Headache Attributed to HIV/AIDS

Headache is reported in over 50% of individuals living with HIV/AIDS and may occur during both acute and chronic phases of infection, often as part of aseptic meningitis or related immune-mediated mechanisms. In the ICHD-3, headache attributed to HIV infection is listed as a distinct diagnostic entity in the Appendix, reflecting its specific clinical context rather than being classified solely as a subtype of headache attributed to infection [5]. In most cases, the headache phenotype resembles primary headache disorders typically bilateral, dull, and tension-type in nature, though migraine-like features are reported in a minority. Clinical studies suggest that headache severity and frequency are more closely associated with indicators of HIV disease activity namely CD4 + T-cell count and viral load than with infection duration or the extent of antiretroviral therapy [2, 5].

Although primary-like headaches are the most common, secondary causes must be carefully excluded, particularly in patients with advanced immunosuppression. Opportunistic infections such as cryptococcal meningitis and cerebral toxoplasmosis remain the most common secondary etiologies of headache in HIV-positive individuals. In such cases, the headache is usually accompanied by focal neurological signs or signs of raised intracranial pressure, and should be classified under the respective underlying condition [5].

#### Headache Attributed to Systemic Infection

Headache is a common but often nonspecific manifestation of systemic infections. Typically, diffuse and moderate to severe in intensity, it may be accompanied by other systemic symptoms such as fever, fatigue, and malaise. In many cases, the headache a tension-type or migraine phenotype, and may also exacerbate underlying primary headache disorders [1, 2, 4].

Epidemiological studies have shown that headache is frequently reported in systemic infections such as influenza, malaria, brucellosis, and leptospirosis, with especially high prevalence noted in certain vector-borne illnesses like rickettsial infections and West Nile virus, where it may occur in up to 80–90% of cases [1].

From a pathophysiological perspective, headache in systemic infection reflects generalized immune activation and inflammatory signaling, in accordance with the shared mechanisms outlined in Sect. 2.2, rather than direct meningeal or parenchymal involvement [1, 2].

#### Post-Infectious Headache Syndromes

While headaches associated with active infections are typically expected to resolve following treatment, a subset of patients develop persistent or recurrent headache in the post-infectious phase, representing a distinct diagnostic and therapeutic challenge. In this context, the headache phenotype may evolve over time and frequently resembles primary headache disorders or chronic daily headache [29].

In ICHD-3 terms, the key practical distinction is whether the headache is persistent, continuing directly from the acute infectious phase without a pain-free interval, or chronic, defined as a new or re-emerging headache following a headache-free period after infection resolution. This temporal distinction is central to classification and follow-up and applies not only to bacterial etiologies but also to viral and fungal intracranial infections. In addition, systemic infections classified under ICHD-3 Sect. 9.2 are similarly subdivided into acute and chronic forms, reinforcing the primacy of temporal evolution over phenotype alone [5].

Clinically, post-infectious headache should be suspected when a new daily or clearly changed headache begins in close temporal relationship to an infection and remains linked to the post-infectious course, particularly after intracranial infections, where symptoms may persist for weeks to months. Systemic infections, including brucellosis, malaria, and chronic viral illnesses, may also give rise to prolonged post-infectious headache, particularly in the setting of sustained immune activation and cytokine-mediated neuroinflammation [2, 5, 6, 30]. Importantly, the ICHD-3 also addresses persistent headache attributed to past intracranial fungal or parasitic infection within the Appendix, underscoring that these entities require specific coding rather than inclusion under generic post-infectious categories [5].

Common diagnostic pitfalls include misclassifying a temporally linked post-infectious syndrome as a primary migraine or tension-type headache without documenting the infectious trigger, overlooking evolving secondary complications such as intracranial hypertension or venous thrombosis, or attributing headache persistence solely to medication overuse without reassessing the original infectious context [2, 6].

**A pragmatic clinical approach includes**:

- confirmation of the temporal pattern (persistent vs. chronic);
- reassessment for red flags and immunosuppression that would warrant diagnostic escalation (neuroimaging and/or CSF evaluation); and.
- if the phenotype remains stable and no red flags are present, symptomatic management while maintaining ICHD-3–consistent classification [2, 5, 6, 25, 31, 32].

##### COVID-19 and Headache

The SARS-CoV-2 pandemic highlighted the high prevalence of headaches related to infection. Headache is a common and sometimes the earliest symptom of acute SARS-CoV-2 infection, occurring in up to 47% of patients. It may precede respiratory symptoms and, in some cases, be the only manifestation of COVID-19. Within the ICHD-3 framework, COVID-19–related headache maps onto existing infection-related headache categories (Fig. 1). The pain is typically bilateral, moderate to severe in intensity, and described as pressing, squeezing, or dull in character. While it often resembles tension-type headache, a subset of patients especially those with a personal or family history of migraine may report a migraine-like phenotype. This includes throbbing quality, photophobia, phonophobia, nausea, and exacerbation with physical activity [33].

In the post-acute phase, headache may persist as part of the long COVID symptom complex. Long COVID is increasingly defined using standardized clinical criteria to capture persistent, multisystem symptomatology extending beyond viral clearance and affecting quality of life for months after infection resolution [34, 35]. Persistent headache affects approximately 8–15% of individuals after COVID-19, lasting for weeks or months beyond viral clearance. These headaches may be daily or intermittent and frequently maintain a migraine-like profile, including sensitivity to light and sound, pulsating pain, and worsening with exertion. They tend to occur more frequently in females and in those who experienced intense headache during the acute illness, although correlations with overall disease severity or hospitalization are inconsistent [33–35]. Recent multidisciplinary post-COVID clinical guidance emphasizes the importance of recognizing persistent headache as part of the broader post-acute symptom complex and supports structured clinical follow-up in patients with protracted symptoms or functional impairment [36].

The pathophysiology remains incompletely defined and is thought to reflect a sustained inflammatory response following SARS-CoV-2 infection, with subsequent dysregulation of pain-processing pathways and involvement of trigeminovascular mechanisms. Proposed contributors include persistent cytokine signaling, altered Angiotensin-Converting Enzyme 2 (ACE2)-related pathways, and immune-mediated processes [33, 37, 38].

##### New Daily Persistent Headache and Post-Infectious Onset

New Daily Persistent Headache (NDPH) is a primary headache disorder characterized by the sudden onset of daily, unremitting pain. While its etiology remains elusive in many cases, infectious triggers particularly viral or flu-like illnesses are frequently reported as precipitating events. Patients often recall the exact date of onset, which may coincide with or shortly follow an acute systemic infection [39].

This post-infectious pattern suggests that NDPH may represent, at least in a subset of patients, an immune-mediated or inflammatory response triggered by infection. Reported triggers include Epstein–Barr virus, cytomegalovirus, and more recently, SARS-CoV-2 [39–42]. The ICHD-3 classifies NDPH under primary headache disorders (Sect. 4.10), although its clinical presentation often overlaps with secondary etiologies [5].

Clinically, NDPH can resemble either migraine-like or tension-type headache phenotypes, with bilateral location, continuous or fluctuating intensity, and variable presence of migrainous features, such as nausea or photophobia. In many patients, the headache begins during or soon after the acute illness and persists beyond the expected resolution phase. Understanding the potential infectious origin of NDPH is essential for diagnosis and tailored management. Although treatment remains challenging and evidence-based options are limited, recognition of the underlying trigger may inform therapeutic strategies [39–41].

##### Clinical Interpretation and Diagnostic Uncertainty

From a clinical perspective, post-infectious headache syndromes constitute a diagnostic gray zone in which secondary infection-related headache and primary headache disorders may overlap [2, 5, 25]. Although headache temporally linked to infection often raises concern for a secondary process, many patients ultimately develop phenotypes indistinguishable from primary headache disorders, most commonly migraine or tension-type headache [2, 25].

The principal diagnostic challenge therefore lies not in recognizing headache during infection, but in identifying when further investigation is required. Persistence of headache after resolution of systemic symptoms, in the absence of neurological deficits, with a stable phenotype and no red flags, generally favors a primary or post-infectious mechanism [2, 5, 25].

By contrast, progressive headache, new neurological signs, altered mental status, immunosuppression, or atypical clinical evolution should prompt escalation of diagnostic workup, even when the initial presentation resembles a primary headache disorder [25].

#### Red Flags and Diagnostic Considerations in Infection-Associated Headache

Accurate recognition of red flags in patients presenting with headache is essential for identifying secondary causes, particularly infectious etiology that may be life-threatening if not diagnosed and treated promptly. The SNNOOP10 mnemonic is a widely adopted clinical tool that supports systematic evaluation of warning signs suggestive of secondary headache disorders, including infections. Fever, neck stiffness, altered mental status, and a history of immunosuppression are considered some of the most predictive indicators of CNS infection [25].

To aid clinicians in identifying infection-related warning signs within the SNNOOP10 framework, Table 1 links each red flag to key infectious differentials and infection-related diagnostic features. This targeted approach highlights infectious causes, such as meningitis, encephalitis, brain abscess, or subdural empyema behind common clinical presentations like fever, altered mental status, immunosuppression, or progressive headache. While the SNNOOP10 tool is traditionally used to screen for all secondary headache causes, this infection-focused adaptation may enhance clinical vigilance in settings where infections are highly prevalent or require urgent recognition.

**Table 1:** SNNOOP10 List of red flags with potential infectious etiologies in secondary headache evaluation

**Sign or symptom**	**Related secondary headaches (most relevant ICHD-3 categories)**	**Potential underlying infectious etiology**	**Infection-related diagnostic features**
Systemic symptoms including fever	Headache attributed to infection or nonvascular intracranial disorders, carcinoid or pheochromocytoma	Meningitis (bacterial, viral), encephalitis (HSV, arboviruses), systemic infections (influenza, COVID-19, dengue)	Acute onset; CSF analysis with pleocytosis; contrast-enhanced brain MR
Neoplasm in history	Neoplasms of the brain; metastasis	Neurotropic infections mimicking tumors (e.g., TB, neurocysticercosis)	Subacute course; focal brain MRI lesions; CSF inflammatory profile
Neurologic deficit or dysfunction (including ↓LOC)	Headaches attributed to vascular/nonvascular intracranial disorders; brain abscess	Bacterial brain abscess, viral encephalitis (HSV, EBV), fungal infections (*Cryptococcus*, *Aspergillus*)	Focal deficits; abnormal brain MRI; CSF pleocytosis ± protein elevation
Onset of headache is sudden or abrupt	Subarachnoid hemorrhage (SAH) and other headaches attributed to cranial/cervical vascular disorders	SAH secondary to infectious vasculitis (e.g., VZV), CVT in systemic infections	Urgent CT; CTA/MRA; CSF analysis if imaging is non-diagnostic
Older age (>50 years)	Giant cell arteritis (GCA), neoplasms, nonvascular intracranial disorders	Herpes zoster, cryptococcosis, TB (in immunosenescence)	Elevated inflammatory markers (ESR/CRP); CSF abnormalities; brain MRI
Pattern change or recent onset of headache	Neoplasms, headaches attributed to vascular/nonvascular intracranial disorders	Acute bacterial meningitis, early viral encephalitis, fungal abscess	Rapid evolution; CSF pleocytosis; early brain MRI change
Positional headache	Intracranial hypertension or hypotension	↑ICP in cryptococcal/TB meningitis, empyema **↓**ICP**:** post-infectious or post-procedural CSF leak (rare)	Opening pressure (↑/↓); brain MRI signs of intracranial hypertension or hypotension
Precipitated by Valsalva (sneezing/coughing/exercise)	Posterior fossa malformations; Chiari malformation	Acute sinusitis with intracranial spread, posterior fossa abscess, CVT	Posterior fossa brain MRI; venography; CSF analysis
Papilledema	Neoplasms, nonvascular intracranial disorders; Intracranial hypertension	Cryptococcal meningitis, TB meningitis, CMV ventriculitis	Opening pressure (↑); brain MRI signs of ↑ICP; CSF analysis
Progressive headache & atypical presentations	Neoplasms and nonvascular intracranial disorders	Subdural empyema, chronic meningitis (TB/fungal), PML	Progressive brain MRI abnormalities; persistent CSF inflammation
Pregnancy or puerperium	Vascular disorders, postdural puncture headache, hypertension disorders (e.g., preeclampsia), CVT	*Listeria* meningitis, puerperal sepsis with CVT	Brain MRI + MR venography;CSF analysis
Painful eye with autonomic features	Posterior fossa/pituitary/cavernous sinus pathology; Tolosa-Hunt syndrome	Cavernous sinus thrombosis (facial/sinus cellulitis), orbital cellulitis	Brain/orbital/cavernous sinus MRI; MR venography
Posttraumatic onset of headache	Acute/chronic posttraumatic headache; subdural hematoma	Post-surgical meningitis, CSF leak with secondary bacterial meningitis	CSF analysis; contrast-enhanced brain MRI
Pathology of the immune system (e.g., HIV)	Opportunistic infections	Toxoplasmosis, cryptococcal meningitis, CMV encephalitis in AIDS	Targeted CSF studies; contrast-enhanced brain MRI
Painkiller overuse or new drug at onset	Medication overuse headache (MOH); drug incompatibility	Drug-induced aseptic meningitis (NSAIDs, IVIG); opportunistic CNS infections due to immunosuppressive therapies (e.g., Cryptococcus, JC virus, CMV)	Case history, Temporal drug association; sterile CSF pleocytosis

An overview of signs and symptoms, their related secondary headache, and distribution in red and orange flags

**Key Abbreviations:***AIDS* Acquired Immunodeficiency Syndrome, *EBV* Epstein-Barr Virus, *ESR* Erythrocyte Sedimentation Rate, *CMV* Cytomegalovirus, *CNS* Central Nervous System, *CRP* C-Reactive Protein, *CTA* CT Angiography, *CVT* Cerebral Venous Thrombosis, *HSV* Herpes Simplex Virus, *ICHD-3* International Classification of Headache Disorders, 3rd edition, *ICP* Intracranial Pressure, *IVIG* Intravenous Immunoglobulin, *LOC* Level of Consciousness, *MRA* Magnetic Resonance Angiography, *NSAIDs* Non-Steroidal Anti-Inflammatory Drugs, *PML* Progressive Multifocal Leukoencephalopathy, *SAH* Subarachnoid Hemorrhage, *TB* Tuberculosis, *VZV* Varicella-Zoster Virus

**Fig. 2:**
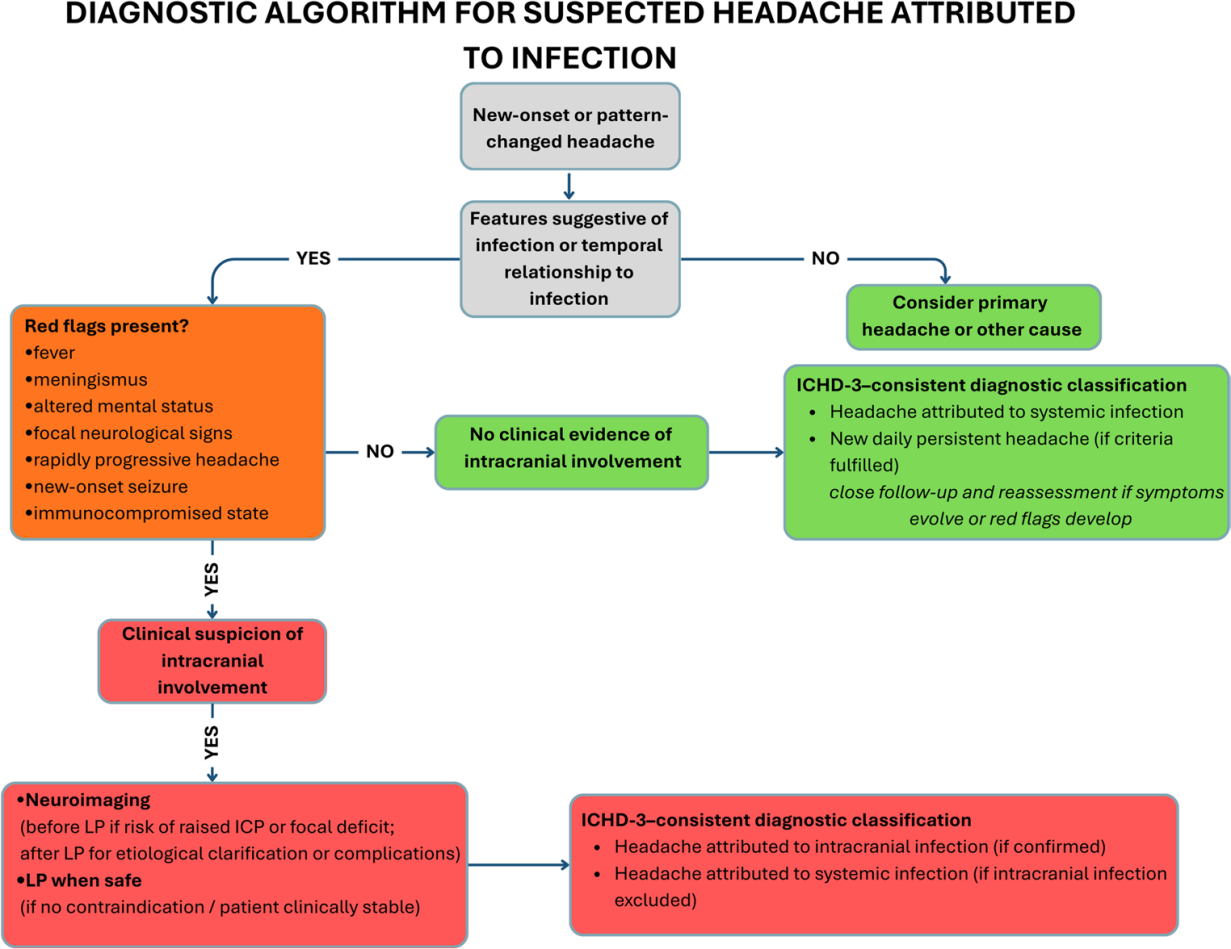
Diagnostic algorithm for suspected headache attributed to infection. This figure presents a stepwise diagnostic approach for patients with new-onset or pattern-changed headache and suspected infectious etiology. The algorithm is guided by clinical features suggestive of infection and red flags indicating possible intracranial involvement. Decisions regarding neuroimaging and lumbar puncture are based on clinical stability and the risk of raised intracranial pressure. In the absence of intracranial involvement, headache attributed to systemic infection or alternative diagnoses should be considered. Final diagnostic classification follows ICHD-3 criteria and the subsequent clinical course. Key Abbreviations: AIDS:Acquired Immunodeficiency Syndrome; CNS: Central Nervous System; ICHD-3:International Classification of Headache Disorders, 3rd edition; ICP: Intracranial Pressure; LP:Lumbar Puncture

Based on these infection-focused red flags, Fig. 2. presents a pragmatic diagnostic algorithm for patients with new-onset or pattern-changed headache and suspected infection.Importantly, the absence of red flags does not rule out serious disease. For example, viral meningitis may present solely with headache and mild fever, while elderly or immunocompromised patients may lack classic signs altogether [2, 25].

## Discussion

Headaches attributed to infection constitute a heterogeneous group of secondary headache disorders encompassing a broad clinical spectrum. Acute presentations such as bacterial meningitis or viral encephalitis are generally more recognized; however post-infectious and systemic infection-associated headache syndromes are often overlooked or misclassified as primary headache types. This diagnostic uncertainty can delay appropriate management and prolong patient morbidity. The ICHD-3 classification offers a valuable framework for distinguishing infectious headache subtypes, including persistent and chronic forms following central nervous system infections. Neverthless, atypical or evolving phenotypes particularly in immunocompromised individuals or those with chronic systemic infections may fall outside conventional diagnostic criteria.

###  Clinical Implications

Patients with post-infectious headache often do not meet the diagnostic criteria for primary headache disorders and may consequently be misdiagnosed. A thorough clinical history focusing on recent infections, combined with cerebrospinal fluid analysis or identification of residual abnormalities on neuroimaging, can provide critical diagnostic clues. Management typically involves symptomatic headache relief using agents such as NSAIDs, triptans, or preventive therapies. In addition, it is essential to monitor for potential complications, including hydrocephalus or cerebral venous thrombosis. Furthermore, addressing commonly coexisting factors such as fatigue, sleep disturbances, and psychological stress is equally important for comprehensive care [1, 2].

## Conclusion

Headache is a common but often underappreciated manifestation of infectious diseases, particularly those affecting the central nervous system. From acute bacterial meningitis to systemic viral infections and post-infectious syndromes, headache can be an early warning sign, a persistent disabling symptom, or the sole clinical presentation.

Recognition of red flags, appropriate classification using the ICHD-3 framework, and a clear understanding of the underlying pathophysiological mechanisms are essential for timely diagnosis and management. Neuroimaging, especially MRI, plays a critical role in identifying complications such as abscesses, empyema, or encephalitis. Moreover, post-infectious headache syndromes, including those seen after COVID-19, require ongoing research attention due to their chronicity and unclear pathogenesis.

Clinicians must maintain a high index of suspicion in patients presenting with new or changing headaches accompanied by systemic or neurological symptoms. A structured, multidisciplinary approach incorporating neurologic, infectious, and imaging evaluation can significantly improve outcomes and reduce morbidity associated with delayed diagnosis of infection-related headache disorders.

## Future Directions

Further studies are warranted to clarify pathogen-specific mechanisms, identify diagnostic biomarkers, and explore long-term outcomes in post-infectious headache syndromes, particularly those following viral and opportunistic infections.

## Limitations

This review is based on a narrative synthesis and does not follow a systematic methodology. Therefore, selection bias cannot be excluded. Additionally, the heterogeneity of included studies and variability in diagnostic definitions may limit generalizability. The review emphasizes clinically relevant patterns but may not capture the full scope of emerging data, particularly for less common pathogens or rare post-infectious headache phenotypes.

## Key References

- Headache Classification Committee of the International Headache Society (IHS). The International Classification of Headache Disorders, 3rd edition. *Cephalalgia*. 2018;38:1–211. 10.1177/0333102417738202.
  - ○ This classification provides the current international standard for diagnosing infection-related headaches, ensuring consistent clinical and research criteria.
- Ramesh R, Ranganathan LN. Headache in Infections. *Curr Opin Neurol*. 2025;38:281–287. 10.1097/WCO.0000000000001348.
  - ○ This recent review offers an updated overview of headache in infections, covering pathophysiology, clinical features, and diagnostic challenges across a broad spectrum of infectious etiologies.
- Do TP, Remmers A, Schytz HW, Schankin C, Nelson SE, Obermann M, Hansen JM, Sinclair AJ, Gantenbein AR, Schoonman GG. Red and orange flags for secondary headaches in clinical practice: SNNOOP10 list. *Neurology*. 2019;92:134–144. 10.1212/WNL.0000000000006697.
  - ○ This article introduces the SNNOOP10 framework, a practical diagnostic tool for identifying red flags, including those associated with infection-related headache.

## Data Availability

No new data were created or analyzed in this study. Data sharing is not applicable to this article.

## Abbreviations

ACE2: Angiotensin-Converting Enzyme 2
AIDS: Acquired Immunodeficiency Syndrome
CGRP: Calcitonin Gene-Related Peptide
CNS: Central Nervous System
COVID-19: Coronavirus Disease 2019
CSF: Cerebrospinal Fluid
CT: Computed Tomography
CVT: Cerebral Venous Thrombosis
DWI: Diffusion-Weighted Imaging
EBV: Epstein-Barr Virus
EEG: Electroencephalography
GCA: Giant Cell Arteritis
HIV: Human Immunodeficiency Virus
HSV: Herpes Simplex Virus
ICHD-3: International Classification of Headache Disorders, 3rd Edition
ICP: Intracranial Pressure
IH: Intracranial Hypertension
IL: Interleukin (e.g., IL-1β, IL-6)
IVIG: Intravenous Immunoglobulin
JC virus: John Cunningham Virus
LOC: Level of Consciousness
LP: Lumbar Puncture
MOH: Medication Overuse Headache
MRI: Magnetic Resonance Imaging
NDPH: New Daily Persistent Headache
NSAIDs: Non-Steroidal Anti-Inflammatory Drugs
PCR: Polymerase Chain Reaction
PML: Progressive Multifocal Leukoencephalopathy
SAH: Subarachnoid Hemorrhage
SARS-CoV-2: Severe Acute Respiratory Syndrome Coronavirus 2
SNNOOP10: Mnemonic for red flags in secondary headache evaluation
TB: Tuberculosis
TNF-α: Tumor Necrosis Factor-alpha
VZV: Varicella-Zoster Virus
